# Automated performance metrics and surgical gestures: two methods for assessment of technical skills in robotic surgery

**DOI:** 10.1007/s11701-024-02051-0

**Published:** 2024-07-27

**Authors:** Rikke Groth Olsen, Morten Bo Søndergaard Svendsen, Martin G. Tolsgaard, Lars Konge, Andreas Røder, Flemming Bjerrum

**Affiliations:** 1 Copenhagen Academy for Medical Education and Simulation (CAMES), Ryesgade 53B, 2100 Copenhagen, Denmark; 2 Department of Urology, Copenhagen Prostate Cancer Center, Copenhagen University Hospital–Rigshospitalet, Copenhagen, Denmark; 3 Faculty of Health and Medical Sciences, University of Copenhagen, Copenhagen, Denmark; 4 Department of Computer Science, University of Copenhagen, Copenhagen, Denmark; 5 Gastrounit, Surgical Section, Copenhagen University Hospital–Amager and Hvidovre, Hvidovre, Denmark

**Keywords:** Automated performance metrics, Surgical gestures, Surgical skills, Robotic surgery, Simulation, Assessment

## Abstract

The objective of this study is to compare automated performance metrics (APM) and surgical gestures for technical skills assessment during simulated robot-assisted radical prostatectomy (RARP). Ten novices and six experienced RARP surgeons performed simulated RARPs on the RobotiX Mentor (Surgical Science, Sweden). Simulator APM were automatically recorded, and surgical videos were manually annotated with five types of surgical gestures. The consequences of the pass/fail levels, which were based on contrasting groups’ methods, were compared for APM and surgical gestures. Intra-class correlation coefficient (ICC) analysis and a Bland–Altman plot were used to explore the correlation between APM and surgical gestures. Pass/fail levels for both APM and surgical gesture could fully distinguish between the skill levels of the surgeons with a specificity and sensitivity of 100%. The overall ICC (one-way, random) was 0.70 (95% CI: 0.34–0.88), showing moderate agreement between the methods. The Bland–Altman plot showed a high agreement between the two methods for assessing experienced surgeons but disagreed on the novice surgeons’ skill level. APM and surgical gestures could both fully distinguish between novices and experienced surgeons in a simulated setting. Both methods of analyzing technical skills have their advantages and disadvantages and, as of now, those are only to a limited extent available in the clinical setting. The development of assessment methods in a simulated setting enables testing before implementing it in a clinical setting.

## Introduction

Patient outcome is affected by the surgeon’s performance; therefore, surgeons must receive relevant training and possess the necessary competencies when operating on patients [1–5]. Virtual reality (VR) simulators allow surgeons to practice their skills in a risk-free environment and receive automated feedback on their performance [6–8]. The optimal way of training is using a mastery learning approach, where all surgeons train to a pre-defined proficiency level which ensures that all surgeons have gained the necessary basic competencies before proceeding to supervised real-life surgeries [9–11]. Proficiency levels on virtual reality simulators have typically been set using simulator-generated automated performance metrics (APM), based on time, instrument movements, and error parameters. However, APM are often presented in a cumulative score with abstract values e.g., instrument path length—left arm (in millimeters), clutch usage (number of times used), number of movements—right instrument (number of times used), etc. [10, 11]. These APM are easy to capture in the simulated setting and often good at measuring skills progression but can be difficult to convert to meaningful feedback, as they say little about the quality of the procedure and the surgical technique used [12–15]. Furthermore, APM are only to a limited extent available during real-life surgeries making them difficult to use for assessment in the operating room.

Therefore, the analysis of surgical gestures was introduced as a new assessment method. Gesture analysis involves breaking down surgery into phases of actions, e.g., ‘needle handling’, ‘grasping’, and ‘suturing’ [16, 17]. Surgical gestures can be used to analyze performance patterns throughout a procedure instead of a cumulative score as with the APM. We can use surgical gestures to provide feedback on how and where in the procedures surgeons can improve [15, 18]. It has previously been used for feedback for suturing models with participants being assessed using surgical gestures and receiving relevant feedback such as “To improve: minimize the number of re-grabs of the needle (< 2 times)” [19]. This method could be available for both simulation-based training and real-life surgeries, presenting a new opportunity for automated evaluation of surgical performance in the operating room. This method is still in the early stages of development as it requires manual interpretation and video annotation of surgical gestures [7, 13, 15, 20].

As both APM and surgical gestures seem to have advantages and disadvantages, we wanted to compare APM and surgical gestures analysis for technical skill assessment in simulated robot-assisted radical prostatectomy (RARP).

## Methods

Messick’s framework was used to describe the validity evidence of the two methods by evaluating the relationship with other variables and the consequences and thereby comparing the two methods.

We compared the advantages and disadvantages of the use of APM and surgical gestures for technical skills as feedback can be difficult for the surgeon using either method. Neither of the methods is available in the clinical setting as the methods for automatic detection of APM and surgical gestures have not yet been developed. With this limitation, only a few studies have assessed the correlation of APM and surgical gestures to patient outcomes, and most studies were performed in urology (Table 1).

**Table 1:** A comparison of the two assessment methods: automated performance metrics and surgical gestures

	Automated performance metrics	Surgical gestures
Ability to measure the progression of surgical skills	• Often used for assessment of performance in simulation-based training and a pass/fail level can be defined • APM have shown a good correlation with clinical skills	• Can assess the experience level of the surgeon and how the procedure was performed • Has yet to be implemented as an assessment tool in the simulated and clinical setting and the measurement of progression of skills has yet to be investigated
Suitability for providing understandable and intuitive feedback	• Provides a cumulative score/measurement for individual parameters • It can be difficult to understand and interpret the clinical relevance of each parameter e.g., moving the right instrument less	• Provides feedback on the progression of the entire procedure by describing the pattern of gestures • An understanding regarding which patterns need to be altered and how they can be changed is still lacking
Feasibility of acquiring during clinical procedures with current technology	• The feasibility is low as the APM are difficult to capture by video analysis and data capture on robot systems is not standard • Further, the APM needs to be analyzed before being presented	• Currently, the feasibility is low as the videos need to be recorded, manually annotated, and analyzed before being presented
Future perspectives	• Automatic capture of APM could be the standard setting in newer, future robotic systems • Other options are machine learning algorithms that automatically extract APM from surgical videos • The assessment of the automatically extracted APM and the correlation to patient outcomes are still needed	• The development of machine learning algorithms will most likely enable automatic annotation of surgical gestures • Further investigation of assessment using the surgical patterns, feedback to the surgeons, and association to patient outcomes are still needed

**Fig. 1:**
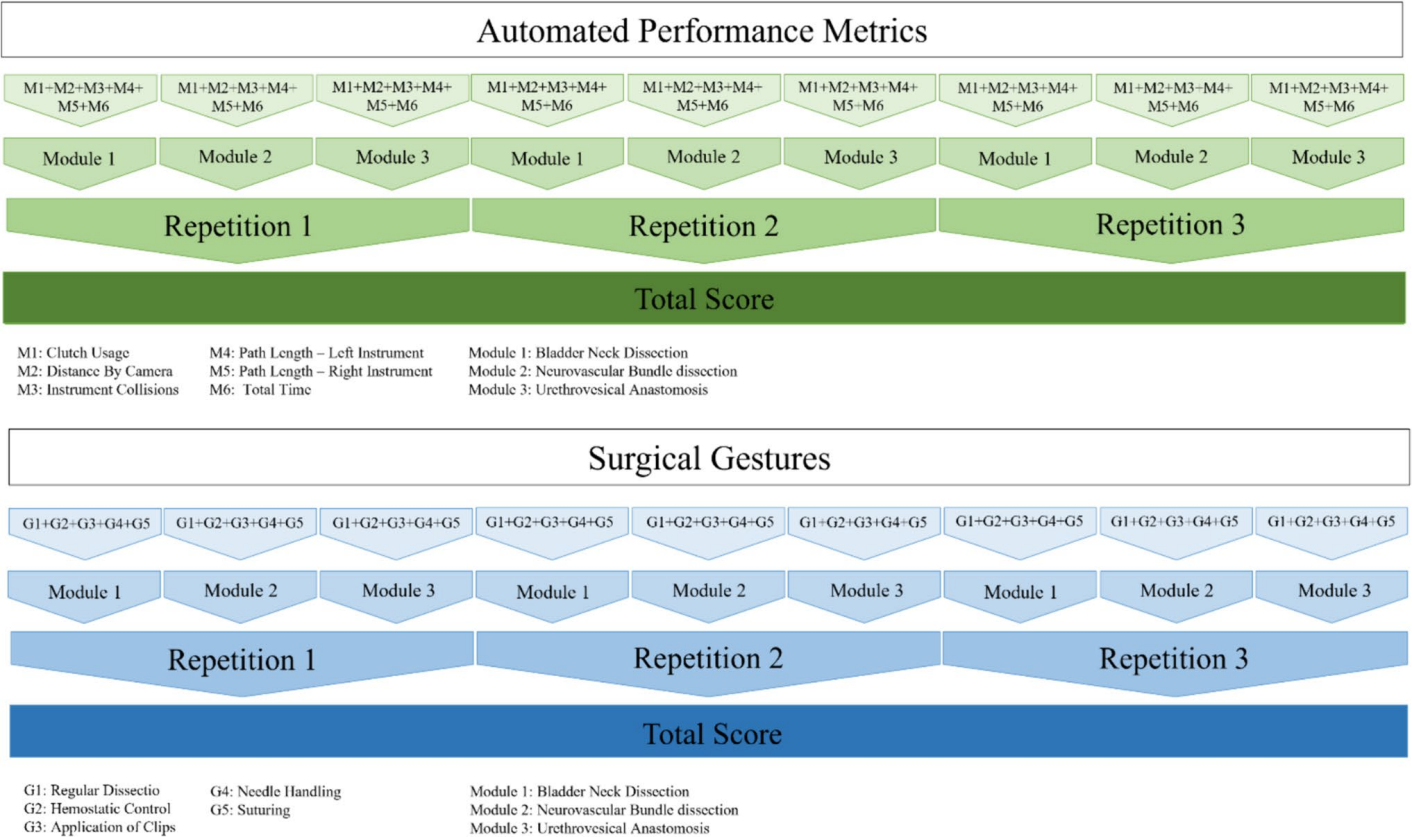
Illustration of how each participant’s total score was calculated

**Fig. 2:**
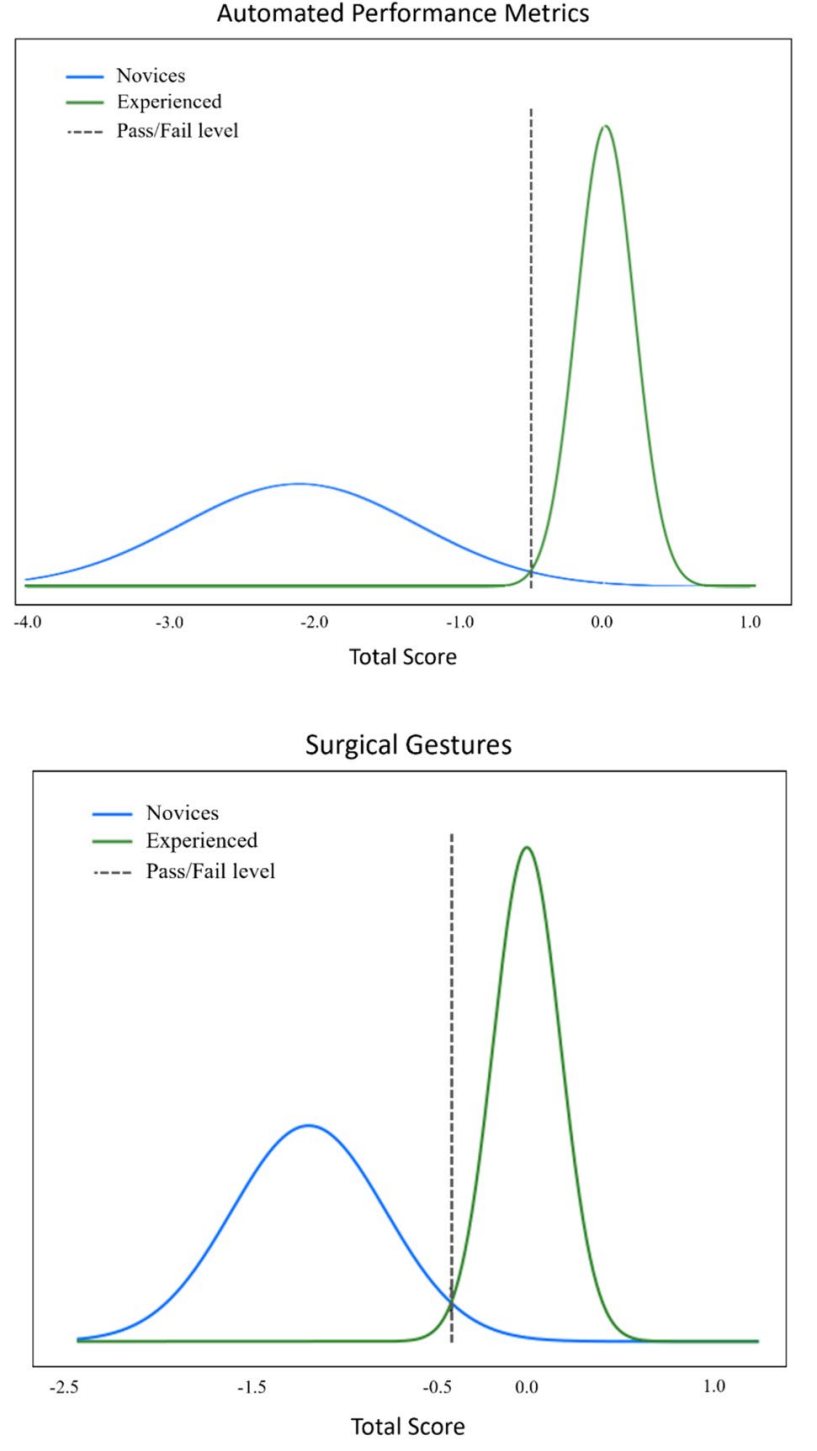
Pass/fail scores for automated performance metrics and surgical gestures for novices and experienced surgeons using the contrasting groups’ method

Ten novice surgeons (assisted to a minimum of one RARP but no other experience with robotic surgery) and six experienced RARP surgeons (performed > 50 RARP) performed simulated RARPs on the RobotiX Mentor (formerly Simbionix, now Surgical Science, Sweden) in a previously published study [21]. APM and videos were automatically recorded for each part-procedure of: bladder neck dissection, neurovascular-bundle dissection, and urethrovesical anastomosis*.* The participants performed each part-procedure three times. In a previous study [21], six of the recorded APM (Table 2) were transformed into *z*-scores, and a composite score was calculated for the six metrics for each of the three modules over the three repetitions giving each participant a total score (Fig. 1). A pass/fail score of −0.51 standard deviations was previously determined using the contrasting groups’ method (Fig. 2).

**Table 2:** A description of the automated performance metrics (APM) and surgical gestures used to assess surgical skills and transformed into *z*-scores to set the pass/fail scores

Automated performance metrics	Surgical gestures
Clutch usage The number of times the clutch is used	Regular dissection Sharp and blunt dissection
Distance by camera The total distance (in mm) moved by the virtual camera	Hemostatic control Whenever the surgeon used energy to control bleeding
Instrument collisions The number of collisions caused by the instrument shaft wrist and jaws colliding with each other	Application of clips Whenever the surgeon uses the clip applier
Path length—left instrument Total distance (in mm) traveled by the left instrument (arm 2)	Needle handling Whenever the needle was handled by either of the instruments and was not in contact with the tissue
Path length—right instrument Total distance (in mm) traveled by the right instrument (arm 2)	Suturing Whenever the needle was in contact with the tissue
Total time Total time elapsed between when the user begins the task and starts moving the instruments, and when the user finishes or exits the exercise

Videos were recorded of each part-procedure on the simulator. The 144 videos were manually annotated with surgical gestures in a previously published study [22]. A total of five different gestures were used: regular dissection, hemostatic control, application of clips, needle handling, and suturing (Table 2). The total times of the surgical gestures were transformed into idle time and active time. Idle time was the time between two phases where no annotations of surgical gestures were made. Active time is the opposite of idle time and was measured as the total duration of phases of surgical gestures. In a previous study [22], the total time for each of the five surgical gestures (Table 2) for each part-procedure was transformed into a z-score, and a composite score was calculated for the five gestures for each of the three modules over the three repetitions giving each participant a total score (Fig. 1). A pass/fail score of −0.4 standard deviations was previously determined using the contrasting groups’ method (Fig. 2).

### Data analysis

To examine the agreement between APM and surgical gestures (*relationship to other variables)*, an intra-class correlation coefficient (ICC1, one-way, random) [23] and a Bland–Altman plot were used using the pass-fail scores for novices and experienced surgeons. The limits of agreement of the Bland–Altman plot were set as the 95% confidence interval for the experienced RARP surgeons. We compared the *consequences* of the two assessment methods by calculating the sensitivity and specificity of the pass/fail levels, e.g., how many novices failed and how many experienced surgeons passed, for APM and surgical gestures.

For data analysis, we used the Python programming language (version 3.10.10, Python Software Foundation, Amsterdam, The Netherlands, https://www.python.org).

### Ethics

The Danish Data Protection Agency approved the study (REG-059-2019 and P-2020-701). The study was deemed exempt from ethical approval by the Danish National Ethics Committee (H-19016423).

## Results

**Fig. 3:**
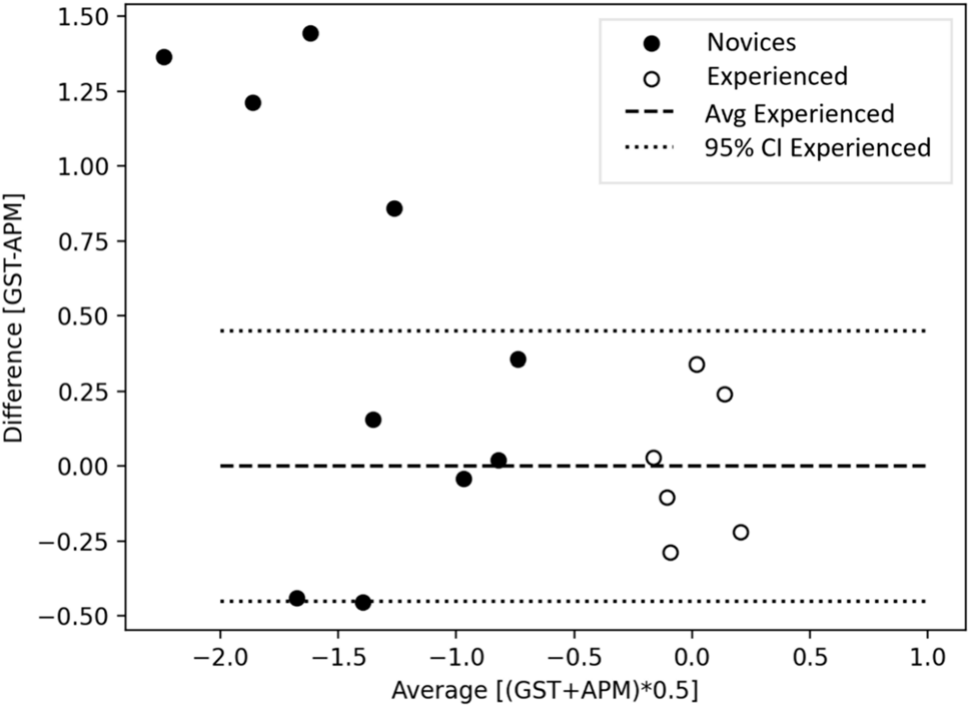
Bland–Altman plot comparing automated performance metrics versus surgical gestures for novices and experienced surgeons to test the agreement between the two methods. *APM* automated performance metrics, *GST* surgical gestures, *Avg* average, *CI* confidence interval

**Fig. 4:**
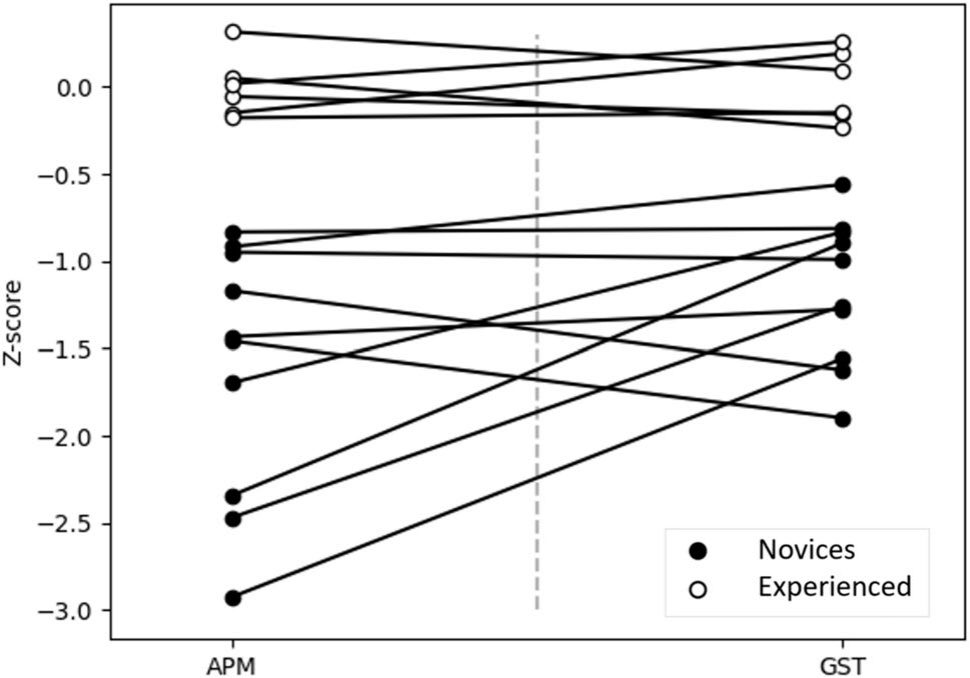
Spaghetti plot where horizontal lines would indicate no differences between z-scores and the steepest lines indicate the largest differences. The outliers from the Bland–Altman plot would therefore be the three lowest points on the APM side. They go from outliers on APM to center locations in the GST observations indicating that the cause of deviance is based on the APM scores, not the GST scores. *APM* automated performance metrics, *GST* surgical gestures

The overall agreement between APM and surgical gestures measured with the intra-class correlation coefficient (ICC) was 0.70 (95% CI 0.34–0.88), which is acceptable (relationship to other variables) [24]. The Bland–Altman plot (Fig. 3) showed an agreement between APM and surgical gestures for all experienced surgeons, with both APM and surgical gestures being effective methods. However, for novice surgeons, the APM score was lower than the surgical gesture scores for 3 out of 10 novices (Figs. 3 and 4). The three lowest combined APM *z*-scores were exceptionally low due to increased instrument collisions for the urethrovesical anastomosis task.

**Fig. 5:**
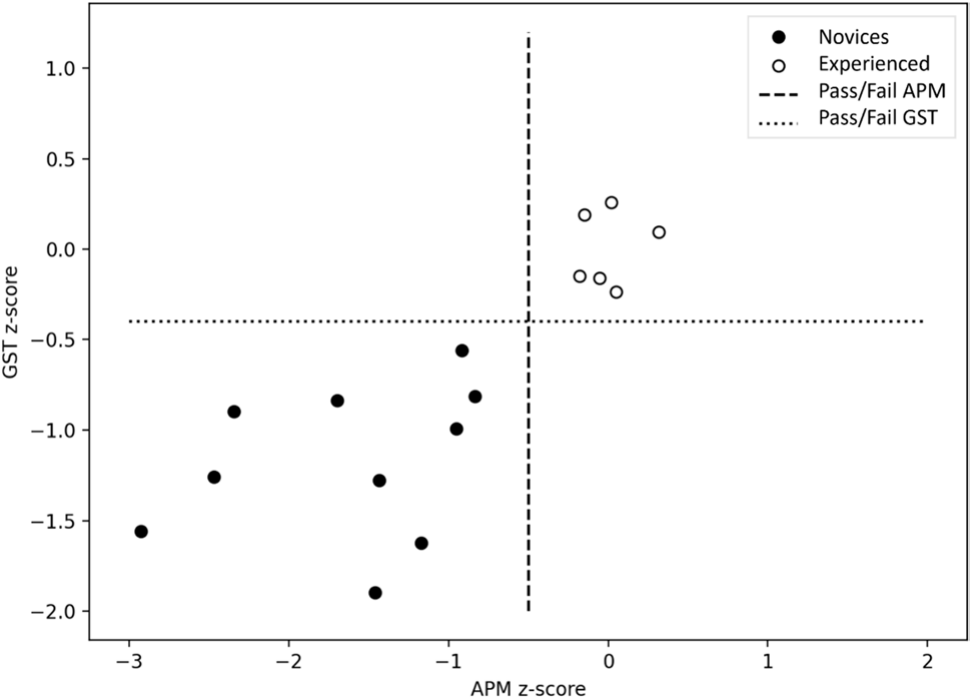
Scatterplot comparing the pass/fail scores of APM versus surgical gestures for novices and experienced surgeons. *APM* automated performance metrics, *GST* surgical gestures

We compared the pass/fail scores of APM and surgical gestures and found that both methods could fully distinguish between novices and experienced surgeons (consequences) (Fig. 5). Both methods could fully distinguish between the skill levels of the surgeons, meaning that all novices failed, and all experienced surgeons passed when using either method.

## Discussion

Both APM and surgical gestures could fully discriminate between novices and experienced surgeons, but there was a greater variance in the pass/fail scores for the novices for the APM scores.

Ideally, surgeons should acquire the initial skills in a simulated setting and practice until they reach proficiency using a relevant pass/fail level. This could be established using APM, rater-based assessment tools, or video analysis [21, 22, 25]. This would ensure that the surgeons have gained the competencies required to proceed to supervised real-life surgeries [10]. Dubin et al. [25] and Jørgensen et al. [26] found that APM and rater-based assessment tools matched for some metrics/items but not for others. We found that the two methods could fully discriminate between experienced surgeons, adding to the validity of evidence supporting both assessment methods. However, we found a greater distribution for the APM scores among the novices compared to the gestures scores, and the two methods disagreed on the skill level of 3 out of 10 of the novice surgeons where one method scored the novices with a higher skill level than the other. These novice surgeons have a low APM score, but they performed at an average level when analyzed using gestures. The three novices all had a higher incidence of instrument collisions in the urethrovesical anastomosis, which is the part of the procedure where the instruments move closely together for needle handling and suturing. This will increase the risk of instrument collisions, but the clinical effect is uncertain, as we can see from the gestures that they manage to perform suturing just as well as their novice colleagues. APM and surgical gestures differ greatly in data type and how the data could be presented to the trainees. APMs are cumulative scores based on movement data from the instruments and the camera and focus on technical skills. In contrast, surgical gestures are specific actions the surgeon performs and are used to analyze the pattern of actions throughout the procedure [10, 11, 15, 18]. This makes surgical gesture analysis more procedure-specific as the workflow will most likely differ between procedures, even though each individual gesture is not procedure-specific [10, 11, 15, 18]. Our previous study showed that the distribution of surgical gestures throughout the part-procedures differed greatly between the novices and experienced surgeons. This could indicate that the two methods measure different aspects of surgical skills, but both can be used to assess competency. Another way could be to provide the trainees with feedback from both APM and surgical gestures. Previous studies have shown that data from both APM and surgical gestures will improve the prediction of AI models on the surgical experience level [15, 27–30]. Trainees could benefit from dual feedback which covers different aspects of technical skills and procedural skills. However, it is still difficult and time-consuming to capture and analyze both types of data.

We have only tested the correlation of the two methods with a limited number of experienced surgeons and did not include intermediate surgeons. We also used data from a simulated setting and not real-life surgery. Previous studies have shown a correlation between APM and surgical gestures on patient outcomes in RARP [18, 31]. The current availability of APM and surgical gestures for real-life surgeries is very limited. In the operating room, it is difficult to find methods for assessment that do not require a more experienced surgeon to be present onsite or spend time reviewing video recordings of the procedure afterward. Onsite clinical assessment is time-consuming for surgeons and subject to bias as the assessor often comes from the same institution as the surgeons in training [32]. APM can be recorded in the clinic using custom recording tools for robotic surgery, but these recorders are both difficult to obtain, and the data can be difficult to interpret [31, 33, 34]. Surgical gestures could be an alternative to unbiased assessment in the clinic. It requires the surgeon to record the procedure and for someone trained in video annotation to analyze the video. Unfortunately, manual annotation of surgical procedures is very time-consuming and not scalable but with the increasing use of artificial intelligence (AI) and automation; in the future, it could be possible to annotate surgical videos with surgical gestures automatically. The greatest force of AI is the ability to detect patterns that we as human evaluators did not consider, e.g., certain part-procedures could be more important for patient outcomes, or novice surgeons tend to have difficulty in different skill sets than expert surgeons. The AI algorithms could provide surgeons with information on their surgical performance levels and, perhaps, the expected patient outcomes. They could also be used for targeted feedback with advice on improving your skills [19]. For smaller procedures such as a simulated suturing task, simple feedback on needle handling or the number of needle punches in the tissue could be provided and most likely enhance the suturing skills of the trainees [19]. Video examples comparing how the trainee performed the suturing compared to an expert could even be provided for extra feedback. For full-length or real-life surgeries, a small deviation in the surgical pattern in one part of the procedure might not influence the surgery or patient outcome, whereas deviations in other parts could. Feedback on every gesture that was not performed optimally throughout an entire procedure might not benefit the surgeon. For the feedback to be targeted and correct, it will require a much better understanding of the patterns of surgical gestures relevant to each procedure. The creation of AI algorithms requires substantial testing before they can be implemented for clinical assessment [7, 13, 20]. Therefore, as of now, it seems that most assessment methods, without the need for expert surgeons, such as APM and surgical gestures, are only available in the simulation-based setting and not for clinical assessment, but this will likely change in the future.

## Conclusion

APM and surgical gestures could fully distinguish between novices and experienced surgeons in a simulated setting. Both methods of analyzing technical skills have advantages and disadvantages and are only available to a limited extent in the clinical setting. This will hopefully change with the technological development of more advanced robotic systems and AI algorithms.

## Data Availability

No datasets were generated or analyzed during the current study.
